# A Case Report of an Early Response to Definitive Chemoradiation for Esophageal Carcinoma Cuniculatum

**DOI:** 10.1155/2020/4674871

**Published:** 2020-04-25

**Authors:** David E. Long, Ahmad Al-Hader, Robert Emerson, Karen Rieger, William Graham Carlos, Hala Fatima, Feng-Ming Kong

**Affiliations:** ^1^Department of Radiation Oncology, Indiana University, Indianapolis, IN, USA; ^2^Division of Hematology and Oncology, Indiana University, Indianapolis, IN, USA; ^3^Department of Pathology, Indiana University, Indianapolis, IN, USA; ^4^Department of Thoracic Surgery, Indiana University, Indianapolis, IN, USA; ^5^Division of Pulmonology, Indiana University, Indianapolis, IN, USA; ^6^Division of Gastroenterology, Indiana University, Indianapolis, IN, USA; ^7^Department of Clinical Oncology, Hong Kong University Shenzhen Hospital, Shehzhen, China; ^8^Department of Clinical Oncology, Hong Kong University Li Ka Shing Medical School, Hong Kong, China; ^9^Department of Radiation Oncology, University Hospitals Cleveland Medical Center, Case Western Reserve University, Cleveland, OH, USA

## Abstract

This case report describes a 63-year-old female with a locally advanced esophageal carcinoma cuniculatum treated with definitive chemoradiation who had a rapid and early response. This case is illustrative of an aggressive behavior with rapid response and rapid recurrence. The cases of esophageal carcinoma cuniculatum as well as the closely related clinical entity of verrucous carcinoma are reviewed suggesting good clinical outcomes after definitive therapy with chemoradiation and/or surgery.

## 1. Introduction

Carcinoma cuniculatum is a rare, well-differentiated variant of squamous cell carcinoma. The first report of esophageal carcinoma cuniculatum suggested similar biologic behavior to verrucous carcinomas, deeply penetrating without lymph node metastases [1]. There is debate whether or not carcinoma cuniculatum should be classified separately from verrucous carcinomas [2]. Both carcinoma cuniculatum and verrucous carcinoma of the esophagus can be challenging to diagnose because biopsies often show nonspecific inflammatory and hyperkeratotic changes [3]. The characteristic histologic finding in carcinoma cuniculatum is the presence of burrowing channels lined by extremely well-differentiated squamous epithelium [4]. A semiquantitative approach has been suggested to increase the specificity of diagnosing esophageal carcinoma cuniculatum. The presence of 7 or more of the characteristic features (hyperkeratosis, acanthosis, dyskeratosis, deep keratinization, intraepithelial neutrophils, neutrophilic microabscess, focal cytologic atypia, koilocyte-like cells, and keratin-filled cyst/burrows) provided 100% specificity for the diagnosis [5]. Due to delays in diagnosis, these lesions are often locally advanced at the time of presentation.

## 2. Case Description

**Figure 1:**
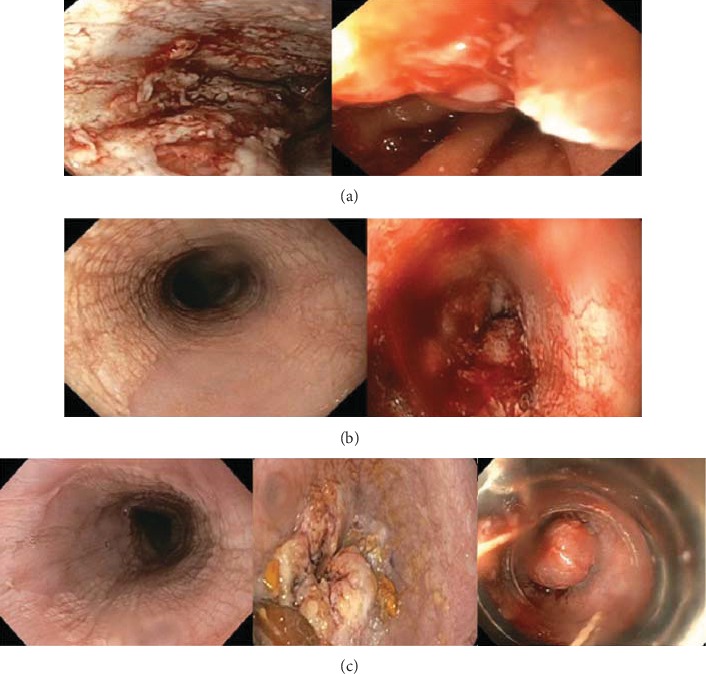
Dynamic endoscopic changes of esophageal mucosa seen on serial endoscopies. (a) From April 2016 demonstrates scattered moderate and severe mucosal changes with white plaques, congestion, erythema, and friability throughout the entire esophagus. Pathology demonstrated squamous mucosa with acute inflammation and Candida esophagitis. (b) From June 2016 noted keratinization in the upper and middle thirds of the esophagus and diffuse inflammation with congestion, erythema, friability, and plaques in the middle and lower thirds of the esophagus. Pathology in both areas showed squamous mucosa with focal erosion with bacterial colonies. (c) From September 2016 noting diffuse severe mucosal changes with congestion, friability, inflammation, fungation, and altered texture in the lower third of esophagus from 30 cm to 40 cm. Pathology from endoscopic mucosal resection was suspicious but not diagnostic of esophageal carcinoma cuniculatum as described in the text.

**Figure 2:**
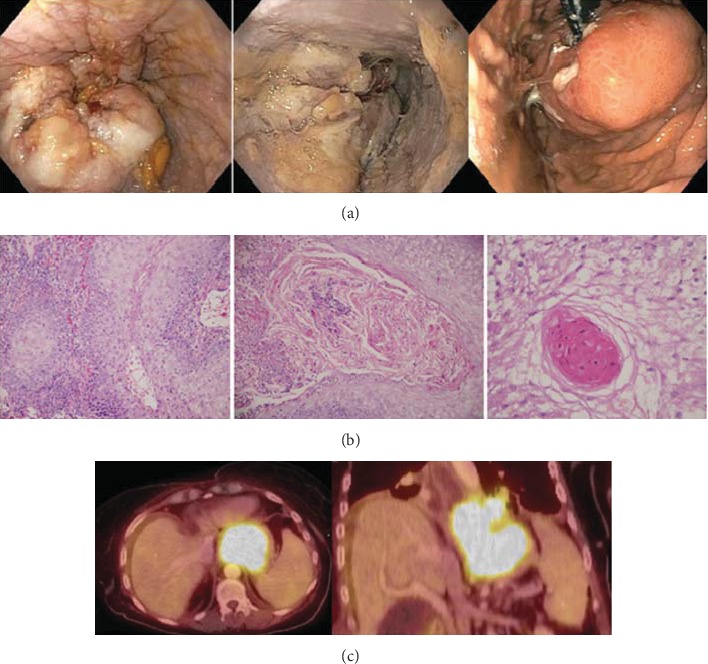
Findings pretreatment. (a) Shows endoscopic views of large, friable, exophytic, and ulcerating mass covered with white exudates in the lower third of the esophagus, gastroesophageal junction, and cardia. (b) Shows pathologic features including (1) a hollow “burrow” with a deep pushing pattern (200x) (2) cyst-like squamous nest filled with keratin and acute inflammatory cells (200x) and (3) keratin pearl in a nest of invasive squamous epithelium. (c) PET/CT shows a large mass in the distal esophagus and proximal stomach without evidence of surrounding adenopathy with mild dilation and likely partial disruption of the distal esophagus.

A 63-year-old woman with a 40-pack-year smoking history, coronary artery disease status post stenting, and a 10-year history of gastroesophageal reflux disease presented with a 4-year history of dysphagia that had worsened significantly in the last 3 months, with an associated 30 lb weight loss. She had 3 prior upper endoscopies over the previous 4 years and the dynamic changes are shown (Figures 1(a)–1(c)). She underwent endoscopy and had diffused severe mucosal changes with congestion, friability, inflammation, fungation, and altered texture in the lower third of the esophagus from 30 to 40 cm (Figure 1(c)). Endoscopic mucosal resection demonstrated squamous mucosa with focal atypia, basal cell hyperplasia, acanthosis, papillomatosis, focal parakeratosis, scattered dyskeratotic cells, fragments of hyperkeratosis, and deeper nests of squamous epithelium with neutrophilic abscesses with occasional multinucleated giant cells. A diagnosis of esophageal carcinoma cuniculatum could not be ruled out. Repeat endoscopy one month later redemonstrated the mass, and endoscopic mucosal resection demonstrated invasive carcinoma with focal “burrowing channels” characteristic of carcinoma cuniculatum (Figures 2(a)–2(b)). Endoscopic esophageal ultrasound demonstrated a large, friable, exophytic and ulcerating mass covered with white exudates in the lower third of the esophagus, gastroesophageal junction, and cardia which was completely obstructing and circumferential extending from 30 cm to 40 cm with extension to the cardia. There was sonographic evidence of invasion into the muscularis propria, and one suspicious 1 cm paraesophageal lymph node was noted, staged uT3N1M0. The mass measured 8.2 × 5.1 × 0.8 cm on PET/CT, and there was no hypermetabolic adenopathy or distant disease (Figure 2(c)). The lymph node was not biopsied on EUS. The patient was admitted to the hospital for dehydration and developed hemoptysis. Repeat CT of the chest at the time noted increased size of the mass with possible invasion of the pulmonary parenchyma. Bronchoscopy with washings was positive for atypical keratinized squamous cells similar to endoscopic resection from the esophagus. She was not felt to be a surgical candidate due to the positive bronchial washings and concern for lung invasion. The final stage was cT4aN0M0, stage IIIA.

**Figure 3:**
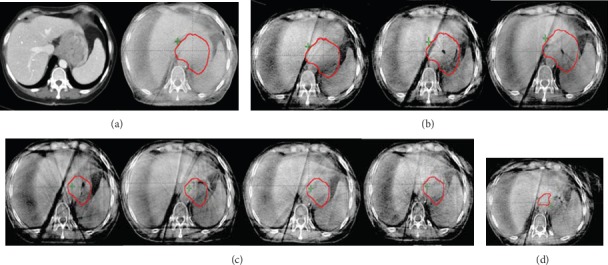
Cone-beam CT (CBCT) changes during treatment. Red outline represents gross tumor volume (GTV). (a) Diagnostic scan pretreatment and CBCT from day 1. (b) CBCT showing treatment changes from weeks 1-3, left to right. Treatment response noted as early as week 2. (c) CBCT for weeks 3-6 after the patient was resimulated for new volumes given marked treatment response. (d) Boost volume based on residual PET-activity.

**Figure 4:**
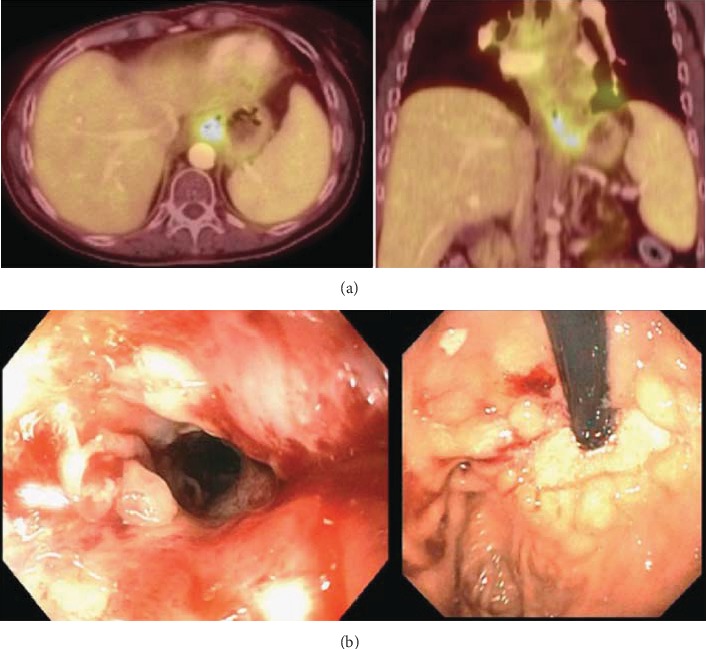
Findings during treatment. (a) PET/CT minimal residual mass with minimal distal esophageal and proximal stomach thickening. (b) Demonstrates postradiation mucosal changes without obvious residual mass and biopsies were negative for residual disease.

The patient was treated with concurrent chemoradiation with weekly carboplatin AUC 2 and paclitaxel 50 mg/m^2^. At the start of treatment, she was only able to swallow ice chips. One week into radiation there was an improvement with the ability to swallow liquids. She was noted to have substantial tumor shrinkage within 2 weeks of starting radiation and was resimulated for adaptive treatment. Cone-beam CT (CBCT) changes throughout treatment are shown demonstrating this rapid response (Figure 3). At week 4 of chemoradiation, she was able to swallow soft food. After 50.4 Gy, PET/CT and endoscopy was performed to assess disease status for surgical evaluation (Figure 4). PET/CT noted an excellent response to treatment with minimal residual mass. There was distal esophageal and proximal stomach thickening. There was a high right paratracheal lymph node which was borderline by both size and activity with an SUV of 3. Endoscopy noted severe mucosal changes with congestion, erythema, friability, granularity, nodularity and ulceration with exudates and desquamation and mucosal sloughing in the lower third of the esophagus from 27 to 36 cm, consistent with radiation changes. There was stenosis at 34-36 cm from the incisors. Biopsies in both the lower esophagus and cardia were negative for malignancy. She was not considered to be a surgical candidate due to her performance status and an additional 5 fractions were given to bring the total dose to 59.4 Gy.

**Figure 5:**
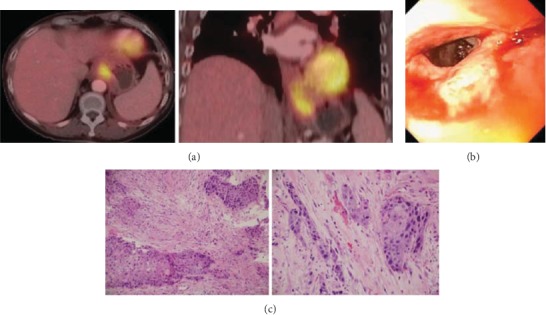
Findings posttreatment. (a) PET/CT noting persistent hypermetabolism and wall thickening in the distal esophagus. (b) Endoscopic finding of severe stenosis in the distal esophagus. (c) H&E stains demonstrating an invasive squamous cell carcinoma (200x and 400x, respectively). This is a higher grade than seen on initial biopsy and may represent a hybrid verrucous carcinoma. There is evidence of radiation treatment effect in the invasive nests.

At 3 months posttreatment, PET/CT noted persistent hypermetabolism and wall thickening in the distal esophagus (Figure 5(a)). She had persistent dysphagia and underwent endoscopy that noted an area of nontraversable stenosis in the lower third of the esophagus (Figure 5(b)). Biopsy at this site was consistent with invasive squamous cell carcinoma (Figure 5(c)). Esophagectomy was discussed with the patient but she refused. She underwent endoscopic debulking followed by systemic chemotherapy (FOLFOX). She had an initial good clinical response but developed progressive disease after 1 month. She did have whole genome sequencing performed including proteomic analysis. There was a somatic mutation in PIK3CA, E542K, making her eligible for Taselisib on the MATCH trial. However, her performance status deteriorated quickly and she was not a candidate. She died of progressive disease 17 months after diagnosis.

## 3. Discussion

This case demonstrates an early and dramatic response to chemoradiation in a locally advanced esophageal carcinoma cuniculatum. These cancers are typically indolent and do not metastasize; however, they can be locally aggressive as in this case. In reviewing the literature, there does not seem to be a clear delineation between esophageal carcinoma cuniculatum and verrucous carcinoma. This case correlated well with the clinical presentation and behavior described in the case series of 11 patients with verrucous carcinomas of the esophagus at Mayo Clinic [3]. The lesion was locally aggressive, had no definitive adenopathy, occurred in the lower third of the esophagus, and was diagnosed in a background of Candida esophagitis. In the Mayo series, 2 patients were treated with neoadjuvant chemoradiation followed by esophagectomy, and 2 patients were treated with definitive chemoradiation. With a median follow-up of 2.5 years (range 1-6 years), there was no evidence of residual or recurrent disease in those patients. This patient unfortunately did have persistent disease versus early recurrence at the time of a 3-month posttreatment assessment. In other series, there were no recurrences in the 6 patients who underwent surgical resection either definitively or after neoadjuvant chemoradiation. In the series of resected esophageal carcinoma cuniculatum from Cleveland Clinic, 2 of the 9 patients died due to perioperative complications [6]. In the remaining patients followed for a median of 84 months, none had evidence of recurrence or metastasis.

The case also demonstrates the typical diagnostic dilemma which occurs with the diagnosis. The largest series on esophageal carcinoma cuniculatum from Cleveland Clinic described the characteristic histopathologic features on esophagectomy specimens given the difficulty with diagnosing this entity on biopsies [6]. These features include hyperkeratosis, acanthosis, dyskeratosis, deep keratinization, intraepithelial neutrophils, neutrophilic microabscess, focal cytologic atypia, koilocyte-like cells, and keratin-filled cyst/burrows. On the initial endoscopic mucosal resection, the patient had 6 of the characteristic histopathologic features suspicious for esophageal carcinoma cuniculatum. It was not until repeat biopsy that the patient met the cutoff of having 7 features including the most characteristic finding of focal “burrowing channels” lined by well-differentiated squamous epithelium. The recognition of the typical histopathologic features can improve the ability to diagnose this difficult clinical entity.

Carcinoma cuniculatum is an extremely rare diagnosis. Verrucous carcinomas in the esophagus are also rare but their treatment in other body sites have been described, including the use of radiation. One of the historical concerns with the use of radiation for this diagnosis was the possibility to induce anaplastic transformation [7]. Anaplastic transformation can occur without radiation [8]. A review of 157 cases of irradiation for verrucous carcinoma of the oral cavity and larynx noted a 7% incidence of anaplastic transformation [8]. The actual rate may be lower than this because sampling errors in biopsy specimens can exist [9]. The invasive squamous cell recurrence in this case did not represent anaplastic change. There was only minimal atypia seen on the initial biopsy which is typical for a carcinoma cuniculatum or verrucous carcinoma, though it is possible there may have been a sampling error (Figure 2(c)). The tumor at the time of recurrence had a higher grade (Figure 5(c)). Based on the tumor behavior in this patient, it is possible that this may have been a hybrid verrucous carcinoma.

## 4. Conclusion

Esophageal cuniculatum and verrucous carcinomas of the esophagus represent rare clinical entities for which optimal management has not been defined. The case reported demonstrates a rapid early response with chemoradiation suggesting it may have a role in the management of this entity, either definitively or in combination with surgery when feasible.
